# Metropolitan Hospital Saturday Fund and Street Collections

**Published:** 1897-11-06

**Authors:** 


### METROPOLITAN HOSPITAL SATURDAY FUND AND STREET COLLECTIONS.

A quarterly meeting of the Board of Delegates of the Metropolitan Hospital Saturday Fund was held at the offices, Gray's Inn Road, W.C., on Saturday, 30th ult., Mr. R. B. D. Acland (chairman of the Fund) presiding. There was a large attendance.

A portrait of Mr. Acland, subscribed for by some fifty members of the Fund, was presented to the Board.

Mr. E. M. Bonus, in making the presentation, said he hoped it would prove a reminder to them all to use the same tact and firmness which had always characterised Mr. Acland's conduct of the business of the Fund. (Applause.)

Mr. Hamilton Hoare proposed that the portrait be accepted by the Board, and hung in the Board room.

This was seconded, and carried unanimously.

The Chairman returned thanks both on behalf of the Fund and of himself.

Street Collections.

A discussion of the question of the Fund's street collection arose out of the report of the Workshop and Street Collection Committee. The committee reported as follows :—

"In consequence of the very serious falling-off in the proceeds of the street collection this year, this committee held a conference on October 5th, in order to discuss the matter and to make arrangements for 1898. Although the shrinkage above referred to may be accounted for in some measure by the institution of the Prince of Wales's Hospital Fund, and the coincidence of the Jubilee festivities, there can be no doubt that there were other causes which conduced to the failure of the street collection of this year. Each local committee was invited to send two representatives to this conference for the purpose of receiving a most important official communication from the Commissioner of Police of the metropolis in connection with the street collection, and of considering the whole matter. Fifty-five representatives attended the conference. After the question had been discussed the following resolution was carried: 'That this meeting is of opinion, in view of the communication from the Commissioner of Police of the metropolis, that it is not necessary to discontinue the street collection; and recommends the Workshop and Street Collection Committee to draw up such regulations as will enable the street collection to be made in such a manner as to meet the requirements of the police and so as not to be an annoyance to the public.'

"In view of the communication from the Commissioner of Police of the metropolis and the consequent necessity for abstaining from using small boxes in the future; in face of the difficulties in obtaining suitable ladies to preside at collecting stations; and in consideration of the expense of the collection, this committee asks the Board of Delegates to decide whether or not the street collection shall be continued."

Mr. W. F. Chalkley (Clapham), in moving the adoption of the report, said that the future course of action on the part of the Board of Delegates as to the management of the street collections was, in face of the letter from the Chief Commissioner of the Police, of the utmost importance, and the committee, therefore, after very careful consideration, had thought it their duty to submit the whole matter to the Board. The letter referred to was dated July 13th last, and was as follows :—

"I am directed by the Commissioner of the Police of the Metropolis to acquaint you that complaints have recently been made to the police by persons who state that they had been annoyed by being followed by collectors for the Hospital Saturday Fund. Although the collectors did not actually solicit money, they were most persistent in following complainants and shaking the money boxes in their faces.

"These complaints are becoming very frequent, and it appears to the Commissioner that the committee of the Fund should take some steps to prevent the importunity of these collectors, for, whilst no one complains of a collector sitting at the street corner to receive money voluntarily given, it becomes a question whether it will not be necessary for the police to remove and treat as ordinary cases of begging the importunate collector who behaves in such a manner as to cause complaints being made to the police."

Mr. G. F. W. Ruppersbery seconded the adoption of the report.

Mr. W. Welch (Woolwich) was surprised at what he had heard. He had never found any difficulty in getting ladies to take charge of stations in his district. To discontinue issuing the small boxes would, in his opinion, ruin the street collection, as for every person who put anything in the large boxes, 20 put into the small ones.

Mr. C. Gregory said he was astonished to hear that the Commissioner of Police should be devoting his time to the consideration of a matter of this sort while there were murderers at large. (Laughter.) This money was collected for the good of humanity, and those who trembled at the sight of the small box ought to be called curs. (Cries of "Order," and laughter.) His idea of the case was that this was the doing of the Charity Organisation Society. They knew that that body was capable of any dirty —— (loud cries of "Order.")

The Chairman: Order, please. It is difficult evidently for us to keep our own house in order, without attempting to keep anybody else's. (Hear, hear, and applause.)

Mr. Gregory, continuing, said they were all cowards to submit to a vague letter like that of the Commissioner of the Police. He was ready to allow his daughter to collect, and, if necessary, to be locked up. (Laughter.) Why not? (Renewed laughter.) If they all trembled at the police he did not. (Renewed laughter.) He moved that the report be not adopted.

The Chairman thought Mr. Gregory had done the greatest possible disservice to the cause of those who held the view that the street collection should be continued, a view with which he sympathised, although he believed it to be a wrong one. It was not the slightest use getting into heroics or hysterics over this question—(laughter) it was a pure matter of business—(hear, hear)—indeed, more than that, it was a matter of statesmanship or policy. (Hear, hear.) Since he had had the honour of addressing them last year on this subject events had marched on considerably. It was not the least use putting their heads in the sand and saying that there was no objection to the street collections. One could not take up a single paper, from the commonest halfpenny to the lordly *Times*, which did not attack the street collections. That meant that there was growing up, and the feeling had increased in volume during the past year, a distaste and dislike of these street collections. The question for them to face was whether they—a body which now occupied a considerable position in the philanthropic world of London—should, for the want of a little foresight, lose that splendid position. (Hear, hear.) Was it worth while even to risk losing it? The question of expense also had to be considered. He pointed out last year that on the then collection of close upon £5,000 the street collection cost 18 per cent. of the amount collected. This year, when the collection amounted to about £2,600, the expenses came to nearly 35 per cent. Were they justified in collecting money at such a cost, when they knew that their Distribution Committee would come down with vigour upon any hospital which dared to collect its money at such a rate? In addition to this they could not get ladies to take charge of their stations. He was not going to ask them to abolish the street collections that night. They all knew his opinion on the subject, but he thought it was of the utmost importance that the matter should be carefully and quietly considered, and so he was going to propose that they appoint a committee to go into the matter and report upon it. In conclusion, he said that the Hospital Saturday Fund was now in a better position than it had ever been before—(applause)—but they must remember that if they ran contrary to public opinion, public opinion would run contrary to them. (Hear, hear.) So, if after careful inquiry it was made perfectly clear that public opinion was adverse to street collections, let them have the courage of their convictions and say that they recognised the force of public opinion. They did not bow to it, but they went with it. Then there was every hope that the great position to which they had attained would be improved. He moved the following resolution :—

"That a special committee be appointed to consider whether in view of the letter of the Chief Commissioner of Police, and the present state of public opinion relative to street collections, it is desirable to discontinue the street collection, and if so, what steps, if any, can be taken to prevent a recurrence of the position referred to in the letter of the Chief Commissioner; that the Secretary and Organising Secretaries do attend the committee; and that the committee do report this day four weeks."

Mr. Gibbs, in seconding, said that those who had gone about the streets during the last year or two must have heard expressions of disgust in reference to street collections. If the Fund did away with the street collection, however, they must find something to take its place, and he asked the delegates to try and find a solution for this. He had been trying in place of the street collections to increase the workshop collections, and in those under his charge he had succeeded so far that the amount collected during the last nine months was rather more than the amount collected during the 12 months of the previous year. (Hear, hear.)

Mr. Hamilton Hoare, in supporting the resolution, asked what the Fund was going to do if they ran counter to the whole Press and to the feelings of sensible men by keeping the street collection? Did they suppose that it would be possible to carry out their scheme in the face of the opposition of the public? Further than this, suppose they did carry on the street collection in the face of this opposition, their expenses would not diminish, but the collection might, and it was possible they might find themselves in the position of not collecting sufficient to pay their expenses. The collection would have to go then whether they liked it or not, and surely it was better that the Fund should take the lead in such a matter, instead of being forced into it. (Applause.)

Mr. W. H. Ross said he had on a previous occasion strongly opposed Mr. Acland's motion, but since then he had looked into the matter, and he had come to the conclusion that it was time to take the question into serious consideration. He strongly supported the resolution.

Mr. W. Newman and Mr. J. B. Clarke also supported the resolution.

Mr. MacGregor did not object to an inquiry being held, although he thought four weeks was too short a time to complete it in. He however did not believe in discontinuing the street collection, as after all the opposition chiefly came from old croakers—persons who would rather write to the police than put a penny in the boxes.

Mr. A. J. Dear thought the local committees should be consulted.

The Chairman agreed that the time should be altered to six weeks, but he could not agree to consulting the local committees. This was a question for the workshops to decide. The point was, were the workshops content to have tacked on to their collection something which the public undoubtedly disapproved of. It was not a question whether the people who made the street collection wished to tack themselves on to the workshops.

The resolution was put and carried *nem con.*, and a committee of twenty was appointed.

The Death of the Duchess of Teck,

A resolution expressing the Fund's sense of the severe loss which the charities of London had sustained owing to the death of the Duchess of Teck was unanimously passed.

The meeting shortly afterwards broke up.

